# A phase I dose-escalation study of neoantigen-activated haploidentical T cell therapy for the treatment of relapsed or refractory peripheral T-cell lymphoma

**DOI:** 10.3389/fonc.2022.944511

**Published:** 2022-11-10

**Authors:** Yuan Chen, Hu Zhao, Jing Luo, Youping Liao, Xu Dan, Guoyu Hu, Weiyue Gu

**Affiliations:** ^1^ Department of Hematology, The Affiliated Zhuzhou Hospital Xiangya Medical College CSU, Zhuzhou, Hunan, China; ^2^ YuceBio Medical Technology Co., Ltd, Shenzhen, Guangdong, China; ^3^ Chineo Medical Technology Co., Ltd, Beijing, China

**Keywords:** neoantigens, haploidentical T cells, adoptive T-cell therapy, peripheral T-cell lymphoma, phase I study

## Abstract

Peripheral T-cell lymphoma (PTCL) is a type of highly heterogeneous non-Hodgkin lymphoma with a poor prognosis and lack of effective targeted therapies. Adoptive T-cell therapy has been successfully used in the treatment of B-cell malignancies. We first used adoptive transfer of haploidentical T cells activated by patient-specific neoantigens *in vitro* to treat an elderly patient with refractory angioimmunoblastic T-cell lymphoma (AITL) in 2017, and the patient achieved long-term complete remission (CR). Here we report on early results from this first-in-human phase 1 clinical trial that aims to assess the safety and tolerability of neoantigen-activated haploidentical T cell therapy (NAHTC) for relapsed/refractory PTCL.

**Clinical trial registration:**

http://www.chictr.org.cn/index.aspx, identifier [ChiCTR1800017440].

## Introduction

Peripheral T-cell lymphoma (PTCL) is a group of highly heterogeneous aggressive non-Hodgkin lymphoma originating from mature T cells or natural killer/T cells in the thymus. The prevalence of PTCL varies greatly between geographical regions. In China, PTCL accounts for approximately 25-30% of all non-Hodgkin lymphoma cases (1, 2), which is significantly higher than that in the US and Europe (10-15%) (3, 4). The standard first-line treatment for PTCL consists of cyclophosphamide, doxorubicin, vincristine, and prednisone (CHOP) or a CHOP-like regimen; however, the outcomes have been disappointing, with a 5-year overall survival (OS) rate of only 20-30% (5). High-dose chemotherapy followed by autologous hematopoietic stem cell transplantation (AHSCT) is recommended as consolidation therapy after complete remission (CR) or partial response (PR). However, Long-term progression-free survival (PFS; 3-year and 5-year) was only 36% to 44% (6, 7). Moreover, approximately one-third of patients cannot undergo transplantation due to early relapse or drug resistance (8, 9). Relapsed or refractory PTCL has been associated with a poor prognosis, of which the median PFS and OS rates was reported to be only 3.1 months, and 5.5 months, respectively (10). Novel drugs such as pralatrexate (11), romidepsin (11) and belinostat (12) were approved for patients with relapsed or refractory PTCL in recent years, but their overall response rates (ORRs) are less than 30%, and their median PFS are less than 4 months. Therefore, it remains a significant clinical need to develop new personalized therapies for PTCL patients which have a high degree of genetic heterogeneity.

Adoptive cell therapy of engineered T cells has been demonstrated to achieve remarkable clinical success in the treatment of B-cell malignancies. This success has led to the broader application of engineered T-cell-based adoptive immunotherapy to other tumors, including T cell malignancies. However, treating T-cell lymphoma with engineered T-cell-based adoptive cell therapy has several unique challenges. First, the shared expression of target antigens between normal T cells and malignant T cells (13, 14) leads to fratricide of therapeutic T-cell products and depletion of endogenous T cells, followed by severe immunosuppression (15). Second, the function and quantity of patient-derived T cells are negatively impacted by tumor microenvironment and previous lines of treatment, limiting the production of engineered T cells from autologous T cells. finally, a real risk of adoptive cellular therapy using autologous products is that circulating malignant T cells are inadvertently mixed with adoptively transferred T cell populations, which may cause malignant T cells to be gene-edited, cultured, and amplified during the *in vitro* manufacturing process, thus artificially accelerating disease progression and the development of drug resistance.

In 2017, We first used adoptive transfer of haploidentical T cells which was activated by patient-specific neoantigens *in vitro* to treat an elderly patient with refractory angioimmunoblastic T-cell lymphoma (AITL) in 2017, and the patient achieved long-term complete remission (CR) (16). This result demonstrated that neoantigen-activated haploidentical T cell therapy (NAHTC) has the potential to overcome the limitations of autologous adoptive T-cell therapy and become a possible therapeutic regimen for PTCL. Thus, this phase I dose-escalation study was conducted on NAHTC to evaluate the safety and efficacy of escalating doses of NAHTC cells for the treatment of relapsed or refractory PTCL. The preliminary results of this ongoing study are reported below.

## Material and methods

### Clinical study and patient information

This is an open-label, dose-escalation, phase I clinical study to evaluate the safety and feasibility of NAHTC in relapsed or refractory PTCL patients. This study was conducted in accordance with the principles of the *Declaration of Helsinki* and approved by the Medical Ethics Committee of the Affiliated Zhuzhou Hospital Xiangya Medical College CSU (Zhuzhou, China). Patients were screened and treated at the Affiliated Zhuzhou Hospital Xiangya Medical College CSU (Zhuzhou, China). Written informed consent was obtained from each patient.

Eligibility criteria included an age of 18 years or older; histologically confirmed PTCL; relapsed or refractory to at least two standard systemic therapy; at least one measurable disease lesion; multiple tumor mutations identified by next-generation sequencing; one haploidentical donor; an Eastern Cooperative Oncology Group performance status (ECOG PS) score of 0 or 1; a life expectancy of more than 3 months; adequate organ function; no evidence of active infections. Key exclusion criteria included known central nervous system diseases; active autoimmune diseases; known positive HIV test; severe active infection; heart, lung, brain, or kidney dysfunction; pregnancy or breastfeeding; participation in another clinical study in the past 4 weeks; and, in the investigator’s opinion, any other condition that makes the patient unsuitable to participate.

### Cell preparation

NAHTC products are manufactured by Chineo Medical Technology Co., Ltd (Beijing), under current good manufacturing practice conditions. NAHTC cells preparation was described in our previous report (16). In short, non-synonymous mutations expressed by individual patients were identified by whole-exome of matched tumor and normal cells and tumor RNA sequencing. We then predicted the binding affinity of peptides to individual HLA molecules and obtained neoantigen candidate epitope sequences. The selected mutations were designed into synthetic RNAs encoding potential neo-epitopes. Peripheral blood mononuclear cells were obtained from the haploidentical donor by leukapheresis, and monocytes were separated by magnetic bead isolation. Monocytes were differentiated into mature dendritic cells (DCs) that were electroporated with these synthetic RNAs and then cocultured with haploidentical T cells isolated using magnetic beads. After priming, neoantigen-specific T cells were screened by using a CD137 magnetic bead antibody, and stimulated to expand for rounds with anti-CD3/CD28 and IL-2 to obtain enough cell dose. The final NAHTC cells were shipped to the clinical site after they met predefined release criteria for count, viability, sterility, and percentage CD3^+^ cells. The manufacturing process of NAHTC cells took approximately 5 to 6 weeks.

### Study design and administration

**Figure 1:**
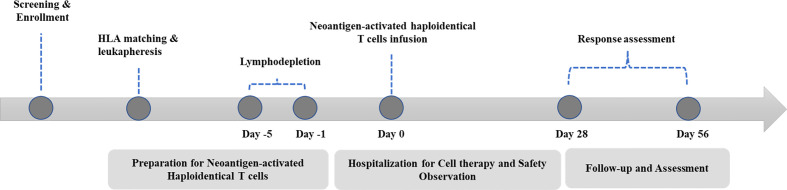
Study treatment schema.

A standard procedure was followed for patient management (**Figure 1**). Basic radiological examinations and blood tests were performed to judge whether the patient was eligible to participate. NAHTC preparation lasted 5 to 6 weeks, and lymphodepletion lasted 5 consecutive days. All patients were given cyclophosphamide 1500 mg/m² per day on days -5 and -4 and fludarabine 25 mg/m² per day on days -3, -2, and -1. A single dose of NAHTC cells was infused on day 0. For safety, an escalating dose was administrated, and three dose levels were set: 2 × 10^7^, 4 × 10^7^, and 8 × 10^7^ NAHTC cells. Each dose level enrolled two patients. Progression to the next highest dose level was allowed after 2 patients were treated on a dose level without a dose-limiting toxicity (DLT). DLTs were defined as any NAHTC-related grade 3 to 4 toxicity occurring within the 30 days of infusion. Dosing of another subject or escalation to a higher dose level will be delayed if qualification of an ongoing toxicity as a DLT is pending.

### Study endpoint

The primary endpoint was safety. The investigators monitored and reported all adverse events that occurred within 40 days after cells infusion. Adverse events were classified according to the NCI Common Terminology Criteria for Adverse Events version 5.0 except for CRS, which was graded according to the CRS grading system developed by Lee et al. (17). The secondary endpoints were response rate. Response was assessed on day 28 after NAHTC infusion according to the lymphoma efficacy evaluation criteria (18). The exploratory endpoints were OS and PFS.

### Serum cytokine analysis

Serum from serial peripheral blood samples collected before and after NAHTC infusion were measure cytokines [interleukin-2 (IL-2), IL-4, IL-6, IL-10, tumor necrosis factor (TNF), and interferon -γ (IFN-γ)] in a single sample using the BD™ CBA Human Th1/Th2 Cytokine Kit II (cat. no. 551809; BD Biosciences) according to the manufacturer’s instructions. The samples were loaded into a FACSCalibur flow cytometer, and the results were plotted and analyzed with FCAP Array™ software.

### TCR sequencing

An ethylenediaminetetraacetic acid–treated blood sample or bone marrow blood sample (2 ml) was collected for total RNA extraction with a Trizol kit. The RNA was used as the starting sample to design oligos in the TCR region for reverse transcription. When reverse transcription progressed to the 5′ untranslated region of the mRNA, reverse transcriptase added several C bases to the 3′ end of the complementary DNA (cDNA) molecule, which paired with the G bases on the added template-switching oligos [this sequence consists of a unique molecular identifier (UMI) sequence and a universal sequence]. Reverse transcription then continued with template-switching oligos as the template, thereby introducing the unique molecular identifier and universal sequences. Next, uracil-DNA glycosylase–degraded template-switching oligos were added, and a PCR product recovery kit was used to purify and recover the cDNA products. A part each cDNA product was used as the template for the first PCR using the 3′ universal sequence of the cDNA as the upstream primer and a downstream primer that targeted the constant region close to the TCR variable region (the downstream primer included a universal sequence). Next, a part of the purified first PCR product was used as the template for a second PCR using the universal sequences at the 5′ and the 3′ ends as the upstream and downstream primers, respectively. After two rounds of PCR, the full-length sequence of the TCR variable region and the introduced unique molecular identifier sequences were obtained, followed by linker addition and library amplification. High-throughput sequencing was performed with the DNBSEQ-T7 sequencing platform (MGI, China). The sequencing process was done at Chigene (Beijing) Translational Medical Research Center Co. Ltd. The sequences were deposited in the NCBI Sequence Read Archive database under BioProject accession number PRJNA853854.

### Statistical analysis

This was an exploratory study, and all analyses were descriptive. The sample size was based on clinical and practical considerations. Descriptive statistics included median (minimum, maximum) for continuous variables and frequency (percent) for categorical variables. Median follow-up time, OS, and PFS were estimated with the use of Kaplan–Meier methods. Analyses were performed with GraphPad Prism 7 software.

## Results

### Patient characteristics

Between August 1, 2017 and May 1, 2020, a total of 7 patients with relapsed or refractory PTCL were enrolled, and 6 patients received an infusion of NAHTC cells. One patient with PTCL-NOS did not receive treatment owing to rapid disease progression before NAHTC cells infusion. The results presented in this study are based on the six patients treated. The median age of the patients was 60.5 years (range, 35 to 75) and the median number of prior chemotherapy regimens was 3.5 (range, 3 to 6). Three patients had been diagnosed with PTCL-NOS, and three with AITL; Three patients had stage III disease, and three have stage IV disease; Of the 6 patients, five patients had refractory to the most recent chemotherapy, and one had had relapsed after conventional chemotherapy and AHSCT. **Table 1** lists the baseline characteristics of all six patients treated.

**Table 1:** Baseline characteristics of patients.

Patient number	1	2	3	4	5	6
Sex	Male	Female	Male	Male	Female	Male
Age (years)	75	58	64	35	52	63
Diagnosis	AITL	PTCL-NOS	PTCL-NOS	AITL	PTCL-NOS	AITL
Number of previous therapies	3	3	4	5	6	3
Number of ASCTs	0	0	0	0	1	0
Ann Arbor stage	III	III	IV	IV	IV	III
ECOG PS	1	0	1	0	1	1
Relapsed/refractory status	refractory to the most recent chemotherapy	refractory to the most recent chemotherapy	refractory to the most recent chemotherapy	refractory to the most recent chemotherapy	relapsed post-AHSCT within 12 months	refractory to the most recent chemotherapy

AITL, angioimmunoblastic T-cell lymphoma; PTCL-NOS, peripheral T cell lymphoma-not otherwise specified; AHSCT, autologous hemopoietic stem cell transplantation; ECOG PS, Eastern Cooperative Oncology Group performance status; NAHTC, neoantigen-activated haploidentical T cell therapy The patient’s order was determined by the time of enrollment and the dose level of NAHTC cells received.

### Safety and tolerability after NAHTC cells infusion

Six patients were enrolled during the dose-escalation phase. The first two patients were treated at dose level 1 (2 × 10^7^ NAHTC cells) and no DLTs were observed. The dose was then escalated to dose level 2 (4 × 10^7^ NAHTC cells) and treated with two patients. As no patient developed DLTs at dose level 2, one patient was enrolled at dose level 3 (8 × 10^7^ NAHTC cells) and experienced a DLT of persistent grade 4 neutropenia and grade 4 thrombocytopenia. As the severe toxicity was observed at dose level 3, it was the decision of the investigators to enrolled one more patient at dose level 2. No additional DLT was observed in this patient. So, dose level 2 was identified as the maximum tolerated dose (MTD) in this study. The only DLT in this study was occurred in patient 5. She was a 52-year-old female with PTCL-NOS. Prior to enrollment on the NAHTC protocol, patient 5 relapsed post-AHSCT within 12 months. As per study design, she was enrolled at dose level 3 (8 × 10^7^ NAHTC cells). The patient had a sustained fever with a maximum temperature of 39.5°C on day 2 after cells infusion. She also experienced grade 3 oral mucositis, grade 4 neutropenia and grade 4 thrombocytopenia. The absolute neutrophil count remained < 500 for 30 days and her platelet count remained < 25 000/mL for 25 days after infusion. However, serum cytokines such as IL-2, IL-4, IL-6, IL-10, TNF, IFN-γ all remained at basal levels after cell infusion. Blood culture and sputum culture were performed and the patient received symptomatic and supportive care, including transfusion, granulocyte colony-stimulating factor, and antibiotic therapy. However, the patient’s condition subsequently worsened with grade 3 elevated alanine aminotransferase, grade 4 elevated aspartate aminotransferase, supraventricular arrhythmia, and prolonged activated partial thromboplastin time on day 20. Blood culture suggested multidrug-resistant *Pseudomonas aeruginosa* infection, and bone marrow examination indicated hematopoietic dysfunction. On day 30, the patient developed severe hypotension and died of severe septic shock, which was considered unrelated to NAHTC treatment.

**Table 2** lists all the adverse events observed in the study. After the infusion of NAHTC cells, no neurological toxicity or CRS was observed in any of the six infused patients. Moreover, no evidence of GVHD was seen. As expected, the most common adverse events were hematologic toxicities. All patients experienced hematologic toxicities with 5 (83%) patients developing grade 3–4 leukopenia, 6 (100%) developing grade 3–4 lymphopenia, 2 (33%) developing grade 3 anemia, and 4 (66%) developing grade 3–4 thrombocytopenia. These are expected toxicities of lymphodepletion chemotherapy. Median duration of high-grade neutropenia was 6 days (range, 1 to 30 days); lymphopenia, 8 days (range, 1 to 33 days); anemia, 13 days (range, 5 to 30 days); and thrombocytopenia, 7 days (range, 1 to 25 days). However, delayed recovery from cytopenia was observed in one patient. Patient 5 experienced prolonged grade 4 neutropenia (30 days) and thrombocytopenia (25 days) after NAHTC cells infusion, despite injection of granulocyte colony-stimulating factor and blood transfusion. The most common nonhematologic toxicity was fever (n=4). Patient 3 had febrile neutropenia and gum pain from days 4 to 6. Patient 4 had a body temperature of 39.8°C on day 2, which lasted for 5 days and chest computed tomography showed signs of lung infection. Patient 5 had a constant fever for 28 days with a maximum temperature of 39.5°C and severe oral mucositis on day 2. Patient 6 had had a fever of 38.8°C on day 3. Intravenous antibiotic treatment and nonsteroidal anti-inflammatory drug were helpful to relieve the fever, but patient 5 had recurrent fever throughout the treatment, and her blood culture suggested multidrug-resistant *P. aeruginosa* infection. Grade 3/4 nonhematologic toxicity was uncommon. All cases of grade 3/4 nonhematologic events occurred in the same patient who experienced the DLT, except there was one case of grade 3 lung infection in patient 4.

**Table 2:** Adverse events in the 6 study patients.

Adverse Event	Grade 1 or 2	Grade 3	Grade 4
	number of patients (percent)		
**Cytokine release syndrome**	0	0	0
**Graft-versus-host disease**	0	0	0
**Neurologic**	0	0	0
**Hematological adverse events**			
Neutropenia	1 (17%)	1 (17%)	4 (67%)
Lymphopenia	0	1 (17%)	5 (83%)
Anemia	3 (50%)	2 (33%)	0
Thrombocytopenia	1 (17%)	2 (33%)	2 (33%)
**Non-hematological adverse events**			
Pyrexia	3 (50%)	1 (17%)	0
Fatigue	1 (17%)	0	0
Muscle weakness	1 (17%)	0	0
Insomnia	1 (17%)	0	0
Nausea	2 (33%)	0	0
Vomiting	1 (17%)	0	0
Gastroesophageal reflux disease	1 (17%)	0	0
Hypotension	0	1 (17%)	0
Superventricular tachycardia	0	1 (17%)	0
Gum infection	1 (17%)	0	0
Mucositis oral	0	1 (17%)	0
Lung infection	0	1 (17%)	0
Elevated aspartate aminotransferase	2 (33%)	0	1 (17%)
Elevated alanine aminotransferase	2 (33%)	1 (17%)	0
Hypoalbuminemia	2 (33%)	0	0
Hypokalemia	1 (17%)	0	0
Hyponatremia	1 (17%)	0	0
Prolonged activated partial thromboplastin time	1 (17%)	0	0

Listed are all adverse events occurred in the 40 days post-infusion. Abnormalities caused by the original disease are not listed. Adverse events were assessed and graded per CTCAE version 5.0; CRS was assessed and graded according to the criteria in Lee et al. (15).

Two of the six patients died including the patient who experienced the DLT. Patient 4 achieved CR within 1 month after cells infusion and had an ongoing CR. This patient died on day 196 and the cause of death was recorded as ‘cardiac sudden death’ which was considered to be unrelated to NAHTC treatment.

### Clinical efficacy

**Figure 2:**
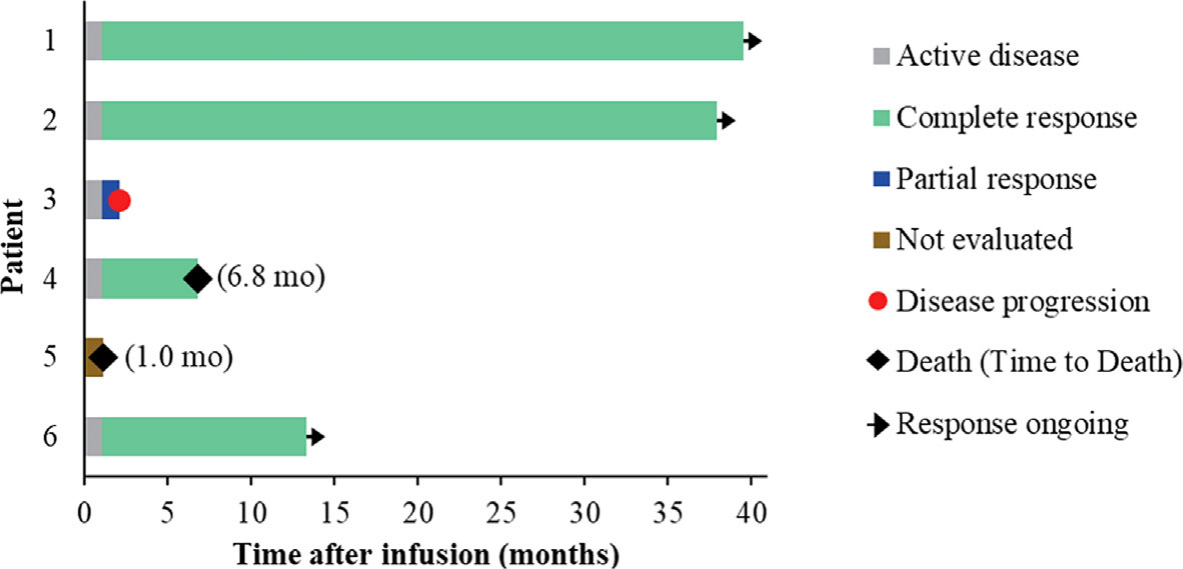
Treatment response of each patient after NAHTC infusion and the duration of response.

Five of the six treated patients were evaluated for treatment response: patient 5 died of severe septic shock before first response evaluation. All five evaluable patients (100%) had an objective response within 1 month of NAHTC cells infusion, which included four CRs (3 with AITL and 1 with PTCL-NOS). Of the four CRs, three had long-term durability with durations of remission of 39.5, 37.9, and 13.3 months, respectively (**Table 3** and **Figure 2**). Clinical response was seen at all dose levels. For the two major PTCL subtypes in the phase 1, AITL subtypes tended to have higher CR rates than PTCL-NOS subtype. All three patients with AITL achieved CR, but only one of the two patients with PTCL-NOS obtained CR, and the other recurred soon after transient PR. In addition, the longest ongoing CR was observed in a patient with AITL.

**Figure 3:**
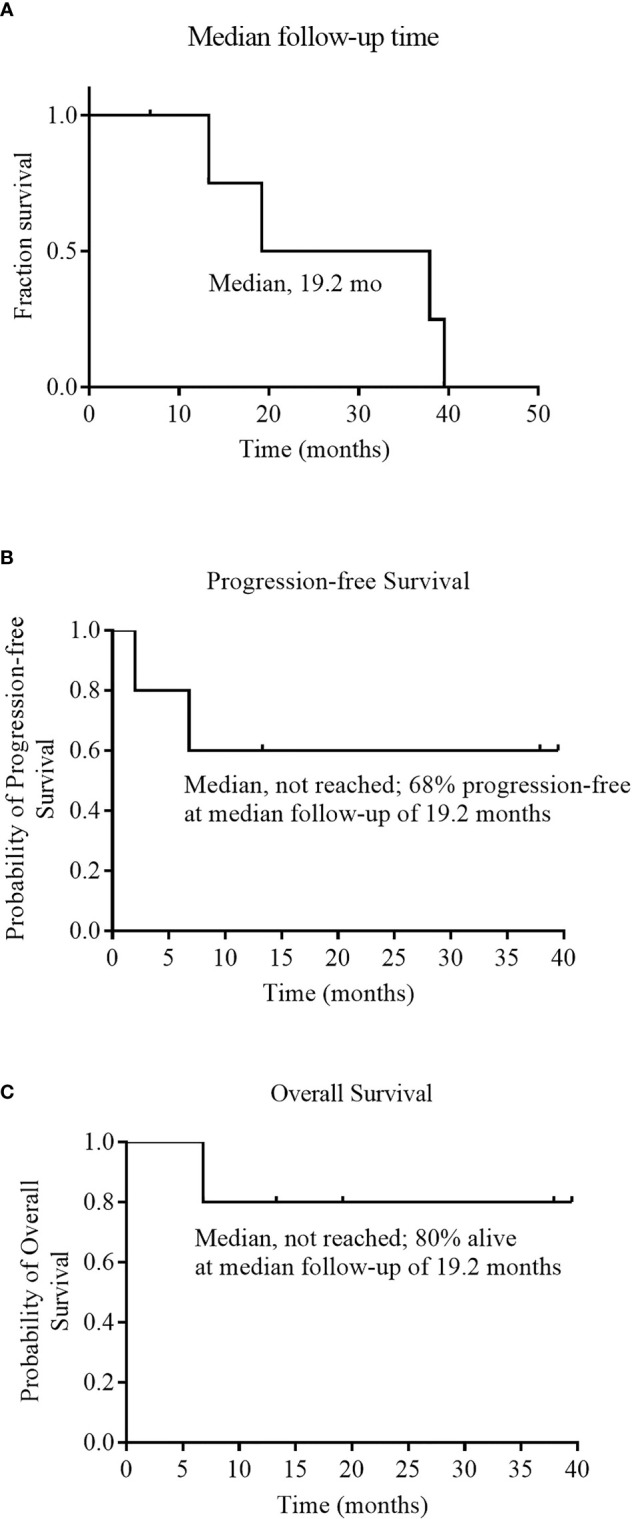
Median follow-up time, Progression-free Survival, and Overall Survival. Panel **(A)** shows the Kaplan–Meier estimate of the median follow-up time among the 6 patients who received an infusion of NAHTC cells. Kaplan–Meier estimates of progression free survival and overall survival among the 5 patients who were evaluated for treatment response are shown in Panels **(B)** and **(C)**, respectively.

As of December 01, 2020, the median follow-up time was 19.2 months (range 6.8 to 39.5) (**Figure 3A**). During the follow-up period, median PFS was not reached among 5 evaluable patients, and 68% of evaluable patients were still progression-free survived (**Figure 3B**). None of the four CRs have relapsed, and only one PR patient relapsed. Patient 2 achieved PR at 1 month after cells infusion; however, the patient had a new mass on the back at 2 months proven as disease progression by imaging examinations and biopsy. Median OS was not reached and four evaluable patients (80%) were still alive at the data cutoff (**Table 3** and **Figure 3C**).

**Table 3:** Clinical efficacy after NAHTC infusion.

Patient Number	1	2	3	4	5	6
Total NAHTC cells infused	2×10^7^	2×10^7^	4×10^7^	4×10^7^	8×10^7^	4×10^7^
Response at 1 month^1^	CR	CR	PR	CR	died on day 30, NE	CR
Response duration (months)^2^	38.5^+^	36.9^+^	1	5.8	NE	12.3^+^
Final outcome	CR	CR	Progressed on day 59	died on day 201	NE	CR

^1^Response at 1 month was defined as the objective response within 1 month of NAHTC cells infusion. ^2^Response duration was from the time of first documented response (complete or partial remission) to the time of progression or death. ^+^means Ongoing clinical response. CR, complete response; PR, partial response; NE, not evaluated.

### Analysis of serum cytokines

**Figure 4:**
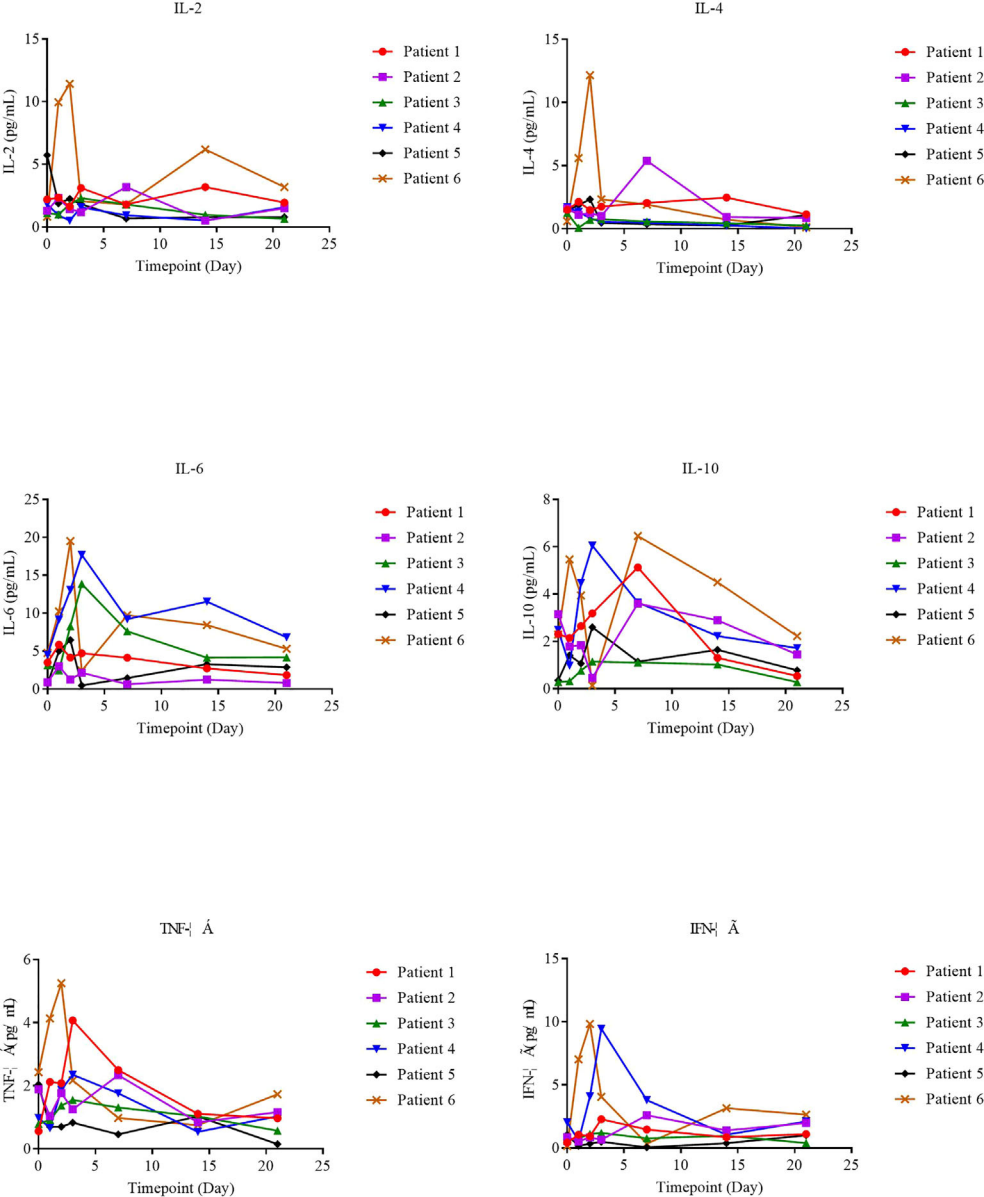
Serum kinetics of a panel of cytokines in 6 patients who received NAHTC infusion.

The supernatants from serial peripheral-blood samples were measured for inflammatory cytokines such as IL-6, TNF-α, IFN-γ, IL-2, IL-4, IL-8, and IL-10, and serum cytokine levels on the day of NAHTC cell infusion (day 0) were taken as the baseline. We found that serum cytokines all remained at basal or low levels on different days after NAHTC cells infusion in all treated patients (**Figure 4**), including those achieving complete and the patient who received the highest dose of 8× 107 NAHTC cells.

### Analysis of TCR sequencing

**Figure 5:**
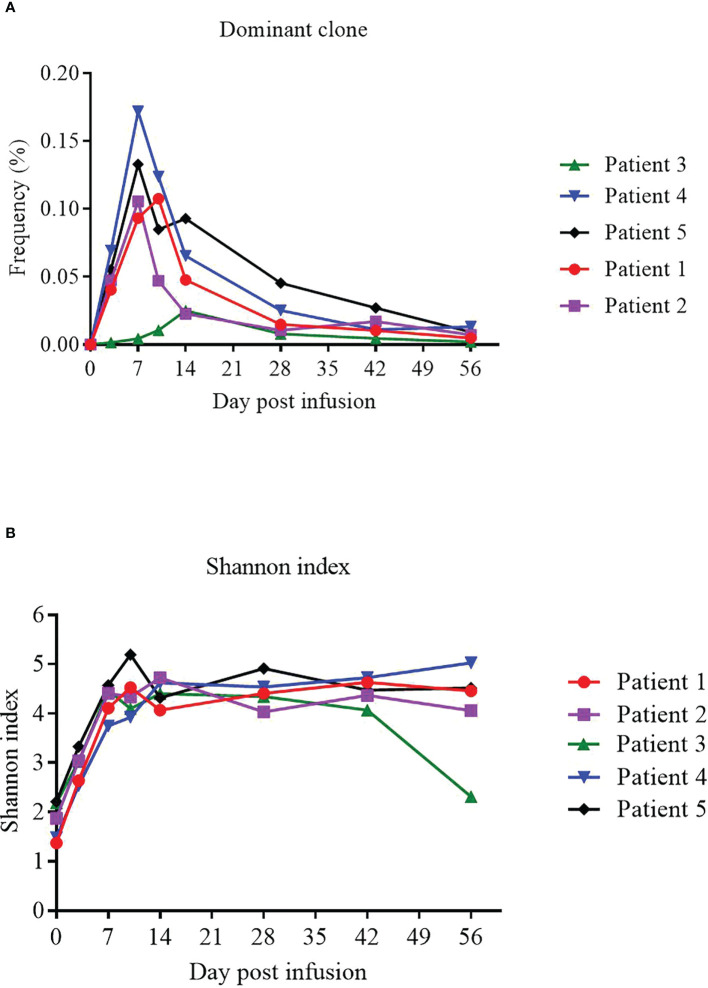
Expansion of the dominant TCR clones from infused NAHTC products and patients’ TCR sequence diversity after NAHTC infusion. **(A)** The peak expansion of the dominant TCR clones appeared in the first 1-2 weeks after infusion. No dose-related difference was observed in the in the peak expansion of the dominant TCR clones. **(B)** Shannon index was significantly increased at all dose levels after cell infusion. However, the Shannon index of the PR patient who relapsed at 2 months after infusion decreased on day 56, which was consistent with the time of relapse. Shannon index treats each TCR subclone proportionally as their relative fraction.

We used TCR sequencing of peripheral blood to track the expansion of the dominant TCR clones from infused NAHTC products and evaluate TCR status of 5 evaluable patients before and after NAHTC cells infusion. We found that the peak levels of the dominant TCR clones appeared in the first 1-2 weeks after infusion, and then dropped rapidly to very low levels or undetectable after 2 months in blood. In this study, the peak expansion of the dominant TCR clones occurred earlier and higher in the four CRs compared with the one PR. Of note, no dose-related difference was observed in the in the peak expansion of the dominant TCR clones (**Figure 5A**). Shannon index which treats each TCR subclone proportionally as their relative fraction was used to measure the TCR sequence diversity in patients’ peripheral blood after cells infusion. We found that Shannon index was significantly increased at all dose levels after infusion. In all four patients with ongoing CR, the Shannon index remained at a high level for 2 months after infusion. However, the Shannon index of the PR patient who relapsed at 2 months after infusion decreased on day 56, which was consistent with the time of relapse. (**Figure 5B**).

## Discussion

To our knowledge, this is the first-in-humans clinical trial demonstrating the anti-lymphoma efficacy of NAHTC in the relapsed or refractory PTCL. The preliminary results show that the NAHTC regimen was tolerable and no CRS or GVHD occurred in any of the patients treated. Four of the five evaluable patients (80%) had a CR. Three of the four CRs had ongoing CR on the date for the last visit. Thus, therapy with NAHTC was safe and could provide durable clinical responses in relapsed or refractory PTCL.

It is well known that CRS is the most common side effect of CAR-T cell therapy. In contrast to CAR-T cell therapy (19), any grade CRS after infusion of NAHTC cells developed in none of the six infused patients. The occurrence of CRS is closely related to the significantly elevated serum cytokines produced directly by rapidly proliferating adoptive T cells bound to target antigens or indirectly by T cell-activated myelocytes (20–22). No significant elevation in serum cytokine levels after NAHTC cells infusion treated was observed in any of the six infused patients. Compared with CAR-T cell therapy with CARs directly specific binding tumor cell surface antigens, NAHTC treatment recognizes tumor neoantigens presented by the major histocompatibility complex of tumor cells, which is a natural T cell activation mode (23–25). This is the possible reason for the lower risk of CRS occurring in NAHTC therapy than in CAR-T cell therapy.

The administered haploidentical T cells may cause life-threatening GVHD in which donor’s transferred lymphocytes recognize allogeneic antigens on the recipient’s tissues. However, we did not see any case of GVHD in this trial. One possible explanation for lack of GVHD is that the presence of NAHTC cells was short. TCR sequencing of peripheral blood showed that the dominant TCR clones among infused NAHTC products were at very low levels or undetectable at 2 months after infusion. In addition, the dose range of NAHTC cells administered on this trial was 2-8 × 107cells, which have been safely used in tumor-infiltrating lymphocytes trials to treat patients with chronic myeloid leukemia and other malignancies (26, 27). The modest cell doses may be another factor to prevent GVHD.

A high incidence of hematologic toxicity was observed in this study, especially grade 3-4 neutropenia and lymphopenia, which we attributed to the lymphodepleting chemotherapy. Severe bone marrow suppression increases the risk of infection, including bacterial, fungal, and viral infections. However, thanks to the use of granulocyte colony-stimulating factor and prophylactic antibiotic therapy, only two patients had severe infection during neutropenic period. Of them, one patient developed a pulmonary infection and recovered after anti-infective therapy, and the other had multidrug-resistant infection and died of severe septic shock. High-grade myelotoxicity indicates that the conditioning regimen of lymphodepleting chemotherapy needs to be optimized. The lymphodepleting chemotherapy regimen doses utilized was selected based on the results of a U.S. National Cancer Institute clinical study (28). Although the dose of fludarabine was similar to that in other studies, the dose of cyclophosphamide utilized here was higher than that used in some recent CAR-T studies (29–31), which may explain the high-grade myelotoxicity observed in this study. Furthermore, recent clinical studies showed that compared with higher dose of conditioning chemotherapy, lower doses of chemotherapy may afford clinical efficacy with an attenuated toxicity profile (32, 33). Thus, the dose of conditioning regimen could be investigated in the future in order to reduce the toxicity of lymphodepleting chemotherapy while ensuring clinical efficacy.

It is not feasible to have a uniform MTD of NAHTC cells for all PTCL patients, because the anti-lymphoma efficacy of NAHTC therapy was not predicted by the number of NAHTC cells infused which have variable expansion *in vivo*. However, high-dose NAHTC cells may increase treatment-related toxicity. Thus, we determined an MTD of 4 × 107 NAHTC cells for future phase II studies.

Among the five evaluable patients, the ORR was 100%: Four (80%) patients achieved CR, and one (20%) patient PR. Responses occurred early and were durable, with three of the four CRs maintaining ongoing CR at 12 months post-NAHTC infusion. These data indicate that NAHTC treatment produce an effective and durable anti-tumor response. Although these results are preliminary and the sample size is small, the activity of single-dose NAHTC treatment is superior to that of the new drugs approved by the FDA for relapsed or refractory PTCL (11, 12, 34). The exact mechanism of this effect is unknown. Lymphodepleting chemotherapy with cyclophosphamide and fludarabine may contribute to the response of NAHTC treatment, even though most patients enrolled were refractory and had received significant amounts of chemotherapy. In addition, clinical response was seen at all dose levels. The frequency and duration of response appeared to be independent of the dose of NAHTC cells infusion, although limited by the small sample size.

Our study cohort consisted of patients with PTCL-NOS and AITL; NAHTC cells might be comparatively more active in AITL compared with PTCL-NOS. CR rate was relatively higher for those with AITL (100%) than for those with PTCL-NOS (50%). Furthermore, the most durable remission was observed in AITL with durations of remission of 39.5 months. Compare to PTCL-NOS, AITL is characterized by high frequencies of overlapping mutations in epigenetic modifiers in neoplastic T cells, including TET2 (80%), RHOA (70%), DNMT3A (33%), IDH2 (20-40%), and CD28 genes (35–37). High-frequency mutations provide more therapeutic targets for personalized tumor immunotherapy, which may be highly relevant to more clinical benefits by NAHTC to AITL patients. These data highlight that given the etiologic differences between the different PTCL subtypes, specific therapies should be prioritized for specific PTCL subtypes. In the future, AITL patients should be the focus of patient selection for NAHTC.

In our study, peak expansion of the dominant TCR clones among NAHTC product was higher and earlier in patients who had a CR than in those who have a transient PR, which suggested that strong NAHTC expansion correlates with anti-lymphoma efficacy. In addition, peak levels of the dominant TCR clones among NAHTC products were not predicted by the dose levels of CAR19 T cells infused which had have different degrees of expansion *in vivo*. Persistence of the dominant TCR clones among NAHTC products was not durable. the frequencies of the most dominant TCR clones contracted rapidly at 1 month after infusion, and t dropped to very low levels after 2 months in the peripheral blood of all patients including four CRs. However, remissions continued in these CRs. These results imply that long-term persistence of NAHTC cells may not be necessary to obtain prolonged CRs of lymphoma. The TCR diversity increased significantly after cell infusion compared with baseline and remained at a high level for 2 months in the four patients with ongoing CR, which suggests that NAHTC treatment may improve the immune status of PTCL patients. The reason for the increased TCR diversity is unknown. We speculate that 1) the TCR clones from the infused NAHTC product increased the TCR diversity, although it is important to note that the dominant TCR clones dropped to a very low level after 2 months of NAHTC cells infusion; 2) neoantigen (dominant epitope)-specific T cells (NAHTC cells) promote tumor cell apoptosis *via* cytotoxic killing, and apoptotic tumor cells release tumor antigens (including concealed epitopes), which may be captured, processed, and presented by antigen‐presenting cells (APCs) again, inducing a response to more epitopes (including concealed epitopes), thereby increasing the breadth of response to tumor-specific T cells; 3) NAHTC treatment improves the patient’s immune response to the tumor microenvironment, which releases T cells and enhances the existing anti-tumor T cell response. These data indicate that, as with neoantigen vaccines (38–41), NAHTC immunotherapy may improve the breadth and clonal diversity of tumor neoantigen–specific T cells, thereby “educating” the autoimmune system to target tumor antigens.

One reason for the success of NAHTC therapy for PTCL may be that such therapy has different mechanisms from all current therapies. First, Tumor neoantigens are *de novo* epitopes derived from somatic mutations, and hence they are not only tumor specific but also highly immunogenic due to lack of central tolerance. Therefore, neoantigen-based NAHTC therapy afford the opportunity to boost tumor-specific immune responses. Second, PTCL have a high degree of genetic heterogeneity which has fundamental implications for the efficacy of immunotherapy. NAHTC cells which is activated by multiple patient-specific neoantigens *in vitro* are likely to target a diversity of malignant clones per patient, thereby addressing tumor heterogeneity, reducing the likelihood of tumor escape by single neoantigen loss and effectively eradicate the disease in its entirety (42, 43), which was confirmed by the finding of this study that multi-year CRs of relapsed or refractory PTCL occurred after NAHTC cells infusion. The above reasons may partly explain why NAHTC therapy produces a potent and durable anti-lymphoma response in PTCL patients who are resistant to current therapies.

This is the first report demonstrating the antitumor efficacy of haploidentical T-cell therapy in patients with relapsed or refractory PTCL haplo-CAR T cell therapy. Haploidentical T-cell therapy has many potential advantages over autologous T-cell One obvious advantage of haploidentical T-cell therapy is T cells from healthy donors, which can ensure their quality and quantity, in contrast to autologous T cells which are often are dysfunctional as a result of exposure to inhibitory tumor microenvironment (44, 45) or to prior intensive therapies. Second, the process of isolating haploidentical T cells form fresh peripheral blood is easy, safe and reliable and also can save time and reduce the costs, so that more patients have access to new treatments. In contrast, the co-expression of many surface antigens between malignant T cells and normal T cells makes it difficult to purify normal from malignant T cells for the manufacture of therapeutic T-cell products. It is technically challenging and costly to obtain enough uncontaminated autologous T cells. More importantly, If the process of T cell purification is not absolute, adoptive transfer of autologous T cells contaminated by malignant T cells would accelerate the progress of disease and the formation of immunotherapy resistance. Last, tumor cells can evade immune surveillance through genetic or epigenetic alterations and become tolerant to the cytotoxic activity of patient-derived T cells (46). In contrast, donor T cells can eliminate tumor cells by targeting tumor-specific antigens (47). These are also the possible reasons for the success of NAHTC therapy.

This study has some limitations. First, it has no control group, and the results may be cofounded by unmeasured variables (such as doctor and patient preferences and unintentional variables) and unintentional selection bias. Second, the sample size is small, and the follow-up time is relatively short. Further research is needed to evaluate the long-term efficacy of NAHTC therapy. Third, the personalized NAHTC therapy has certain inherent limitations, such as complex and time-consuming of NAHTC cell manufacturing, the considerable cost of the processes. Last, the current conditioning regime has high hematologic toxicity, which must be optimized.

In summary, this study demonstrates that NAHTC therapy is safe and effective for the treatment of relapsed or refractory PTCL and does not cause CRS or GVHD. This novel personalized immunotherapy overcomes several key issues that limit the development of T-cell therapy for T-cell lymphoma and has the potential to eradicate conventional drug resistance cells of-cell lymphoma which may change the treatments of some refractory PTCLs. While the results are promising, randomized multicenter studies are needed to further evaluate the anti-tumor activity of this novel adoptive T-cell immunotherapy therapy. Moreover, the mechanisms of NAHTC treatment still need to be investigated.

## Data availability statement

The datasets presented in this study can be found in online repositories. The names of the repository/repositories and accession number(s) can be found below: https://www.ncbi.nlm.nih.gov/, PRJNA853854.

## Ethics statement

The studies involving human participants were reviewed and approved by the Medical Ethics Committee of the Affiliated Zhuzhou Hospital Xiangya Medical College CSU (Zhuzhou, China). The patients/participants provided their written informed consent to participate in this study.

## Author contributions

The conception and design of the study: GH, WG, YC. Acquisition of data: GH, YC, HZ, CS, JL, YL. Analysis and interpretation of data: YC, DX. Drafting or revising the manuscript: YC, GH. All authors contributed to the article and approved the submitted version.

## Conflict of interest

Authors WG and XD are employed by Chineo Medical Technology Co., Ltd and YuceBio Medical Technology Co., Ltd, respectively.

The remaining authors declare that the research was conducted in the absence of any commercial or financial relationships that could be construed as a potential conflict of interest.

## Publisher’s note

All claims expressed in this article are solely those of the authors and do not necessarily represent those of their affiliated organizations, or those of the publisher, the editors and the reviewers. Any product that may be evaluated in this article, or claim that may be made by its manufacturer, is not guaranteed or endorsed by the publisher.

## Supplementary material

The Supplementary Material for this article can be foundonline at: https://www.frontiersin.org/articles/10.3389/fonc.2022.944511/full#supplementary-material
