# Supplementary figures and images for: A phase I dose-escalation study of neoantigen-activated haploidentical T cell therapy for the treatment of relapsed or refractory peripheral T-cell lymphoma
Source: Front Oncol. 2022 Nov 10;12:944511. doi: 10.3389/fonc.2022.944511 (PMC9684663; doi:10.3389/fonc.2022.944511)

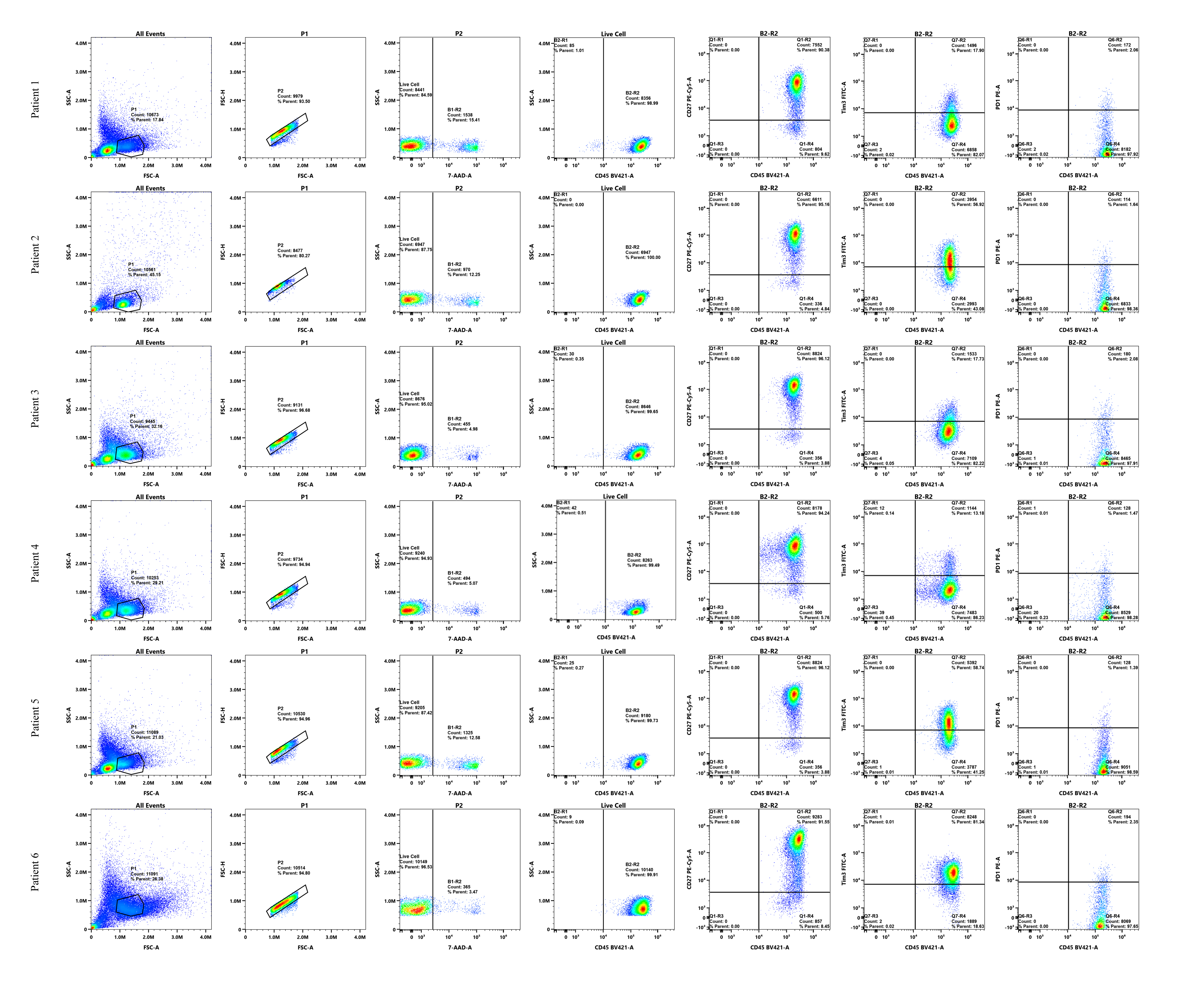

Supplement: Supplementary file 1 [file Image_1.jpeg]
